# Effects of Fiber Arrangement on Flow Characteristics Along a Four-Fiber Element of Fiber Extractors

**DOI:** 10.3390/mi16040425

**Published:** 2025-04-01

**Authors:** Oluwaseyi O. Ayeni, Holly A. Stretz, Ahmad Vasel-Be-Hagh

**Affiliations:** 1 Department of Chemical Engineering, Tennessee Technological University, Cookeville, TN 38501, USA; ayenioluseyio@gmail.com; 2 Department of Mechanical Engineering, University of South Florida, Tampa, FL 33620, USA; vaselbehagh@usf.edu

**Keywords:** process intensification, advanced manufacturing, microfluidic, immiscible flow

## Abstract

Fiber extractors, as process-intensified equipment, facilitate many applications, such as the purification of oils. The development of high-fidelity computational models is crucial to optimize the design. However, simulating microscale flows around tens of thousands of microfiber arrays is computationally unfeasible. Thus, it is necessary to identify smaller elements, consisting of only a few fibers, that can represent flow within massively arrayed fiber extractors. This study employed computational fluid dynamics to investigate different configurations of four-fiber elements to achieve this aim. Following previous modeling featuring flow around only one fiber, the goal was to understand how variations in inter-fiber distances affect the phase structures of a corn oil/water mixture, the steady-state interfacial surface area per unit of fluid volume, and the pressure drop along the flow direction. The study explored various total and relative flow rates and contact angles. The research characterized the flow as semi-restricted annular, noting the influence of neighboring fibers on phase complexity. The inter-fiber distance played a crucial role in generating high interfacial areas and reducing pressure. The chaotic nature of the slug interfaces facilitated intermixing between flows along different fibers. Interestingly, the specific interfacial area reached an optimum when the inter-fiber distance was between 10 and 50 μm.

## 1. Introduction

Bioprocessing relies significantly on purification processes, one of which is liquid/liquid extraction (one example: aqueous two-phase extraction for high-value pharmaceuticals) [1,2,3,4,5,6,7,8]. The early development of large interfacial surface areas affects the efficiency of such separation mechanisms. Conventional contactors such as mixer settlers, centrifugal contactors, agitated columns, and static mixers can facilitate relatively large interfacial areas. However, the complex hydrodynamics involved in typical contactors create a challenge in defining the interfacial surface areas [9,10].

Microfluidic devices, such as micro-structured reactors (MSRs) [11], have emerged as industrially viable alternatives to facilitate large interfacial surface areas, resulting in higher energy efficiency and product purity and lower waste, as desired by the biofeedstock process industry (example: purification of vegetable oils [7]). However, parallel processing of microfluidic tubes is challenging due to the complexities of scaling up to commercial throughput. Some approaches involve bundling parallel microfluidic tubes in order to increase throughput; however, process control of such bundles (when scales are at thousands or more of microfluidic channels operating in parallel) remains a challenge [11,12,13]. Bundled designs require the numbered microchannels to be separated, which makes it complex to control and maintain the uniformity of flow and heat transfer across the device. In addition, bundled designs demand the construction of special junctions [14,15].

Fiber extractors, FEs, have emerged as a novel and viable solution to these challenges as they preserve the sustainable benefits of microfluidics processing while working readily with current infrastructure for process control and scale-up [16,17,18,19]. (Note that a different type of processing, contactors with parallel hollow fibers where fluids flow through the fiber hollow, is being reported for areas such as carbon capture [20]. The fluid flows in these contactors are not the same as in the present work. Flows inside a hollow fiber experience “wall” constraints. Flows around a fiber do not experience “walls” in every direction radially outward, thus the need for modeling.) FEs can feature ~100,000 microfibers, even in a pipe as small as one inch in diameter. Due to massive arrays of microscale passages forming between these fibers for the fluids to flow through, FEs can achieve high production rates. Further, heat transfer and even electrification of a reaction become facile in process control by using metal fibers, such as steel fibers. Ayeni et al. have provided an expanded review of the state of the art of FE processing [21].

Designing an efficient FE requires a thorough understanding of its flow characteristics. Previous studies have primarily concentrated on varying process parameters to examine extraction efficiencies and overall mass transfer coefficients [22,23,24,25]. Consequently, there is a dearth of knowledge about flow dynamics through FEs, especially regarding the development of phase structures within these devices. Gaining this knowledge through experimental methods is quite challenging due to the large number of microfibers in FE devices and the difficulty in visualizing the microscale phase structures that form within the space between these fibers [21]. An alternative approach is using computational fluid dynamics (CFD). However, simulating multiphase flows through thousands of microscale fibers also poses significant challenges, explaining why, to our knowledge, no multi-physics model has ever been developed that effectively captures the unique flow characteristics of FEs.

Simulating an entire fiber extractor (FE) containing thousands of microfibers is computationally impractical. However, we can gain insights into the flow characteristics within such devices by simulating the flow around smaller components, referred to as a (potentially) representative volume element (RVE) hereafter. While these smaller simulations may not fully represent the entire system, they can still provide valuable information about flow along the microfibers. In a recent study, we reported a simulation of a core, one-fiber scenario where the RVE was conceptualized as a micron-scale rectangular area with a single 50 µm diameter fiber located at its center [21]. Two different materials, corn oil, and water, flowed along this fiber through the RVE, with the fiber treated as a wetted wall and the RVE sides functioning as symmetry planes. While the earlier one-fiber study shed light on how the phase structures formed and interacted along a single fiber, it lacked the influence of neighboring fibers.

Therefore, the current study builds upon that idealized one-fiber RVE [21] to include four fibers to understand how neighboring fibers potentially influence the flow and phase structures. Similar to the previously reported single-fiber case, this study also employs water and corn oil feeds as the working fluids, a situation commercially relevant to biodiesel production and vegetable oil refining. This study examines the effects of inter-fiber distance (i.e., packing density), contact angle, and flow rate on the evolution of phase structures, pressure drop, and specific interfacial areas. Specific interfacial surface area is a volume-normalized surface area that facilitates a fair comparison between interfacial surface areas produced within various representative volume elements. Interfacial surface area can be employed in mass transfer calculations to predict extraction efficiencies. The surface area is also useful for designing downstream coalescence mechanisms.

This study modeled the fluid environment via a finite element method (FEM), the details of which are presented in Section 2. The results of this computational analysis are then presented and discussed in Section 3, followed by a series of concluding remarks and recommendations for future work.

## 2. Materials and Methods, Numerical Analysis

### 2.1. The Configurations

**Figure 1:**
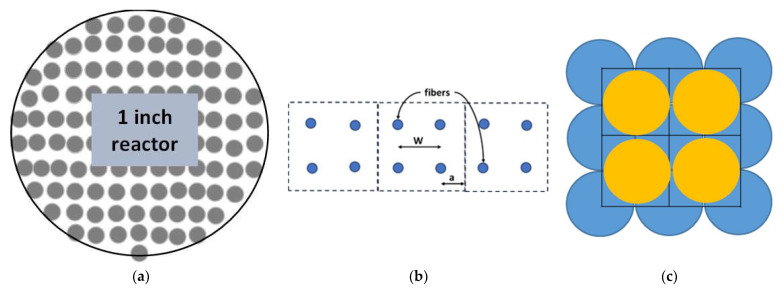
(**a**) A representation of a 2D cross-section of a hypothetical fiber extractor vessel. (**b**) Tessellating square-shaped RVEs containing four fibers, with W representing the inter-fiber distance and a being one-half of W. (**c**) Pictorial representation of channels or pathways formed by the fibers.

Figure 1a shows a schematic of a 2D section of a fiber extractor’s vessel when packed with arrayed fibers. The simulations reported in this study explored the flow and interactions of water and oil along representative volume elements (RVEs) that include four microfibers to take a step toward developing a better understanding of flow around solid fibers within fiber extractors. The study employed square RVEs comprised of four equally spaced fibers with a characteristic inter-fiber distance, “W” (Figure 1b). Another characteristic length is the distance between the fiber’s edge and the RVE’s edge, shown as “a” (Figure 1b). At the four outer edges of the RVE, symmetry conditions were applied. The inter-fiber distance was then varied across various cases using two approaches, i.e., constant dimensions, hereafter “CD”, and constant voidage, hereafter “CV”, resulting in inter-fiber distances ranging from 40 to 114 μm and fiber diameters between 5 and 59 μm. In all the cases studied, the fiber length was fixed at 1 cm, which was determined by the required computational resources. The high-fidelity simulations included in this research demanded several thousand CPU hours per case when utilizing a high-performance computing cluster. The term “voidage” is used to mean the volume around the fibers inside of the RVE available for fluid flow.

Using method “CD”, the dimensions of the RVE were held constant while changing the diameter of the fiber from 40 to 70 μm (Table 1). Consequently, the inter-fiber distance and voidage also varied with these changes. If these variations were configured for a hypothetical extractor with a specific vessel diameter, all cases generated using the CD method would result in the same number of fibers (e.g., 87,700 fibers in a one-inch-diameter vessel, as shown in Table 1). Therefore, all scenarios created through the CD method can be considered as belonging to the same FE, sharing the same vessel diameter and the same number of fibers, but differing in fiber diameters and, consequently, in the dimensions of the flow pathways that form between the fibers (Figure 1c). Note that the fluids flowing in these pathways can intermix more with larger void space because these pathways are only semi-restricted, i.e., touching fiber surfaces but not on all sides. Also, note that for method “CD” to be a valid packing configuration, the four fibers had to fit inside the RVE without touching. Therefore, an 80 μm fiber diameter would be invalid. Table 1 summarizes the different RVE geometries modeled using method CD and presents how the changes in fiber diameter resulted in various packing densities, void percentages, fiber counts, and inter-fiber spacings.

In method “CV”, on the other hand, the voidage was held constant at 66%, identical to that of the previously studied single-fiber case [21]. Thus, the RVE’s size changed when changing fiber diameters to maintain the same voidage. Also, keeping the voidage constant means that the fluid volume remains unchanged for various fiber diameters, although inter-fiber distance changes. Unlike the method “CD”, this method could employ any fiber diameter without fiber surfaces coming into contact (Table 1).

**Figure 2:**
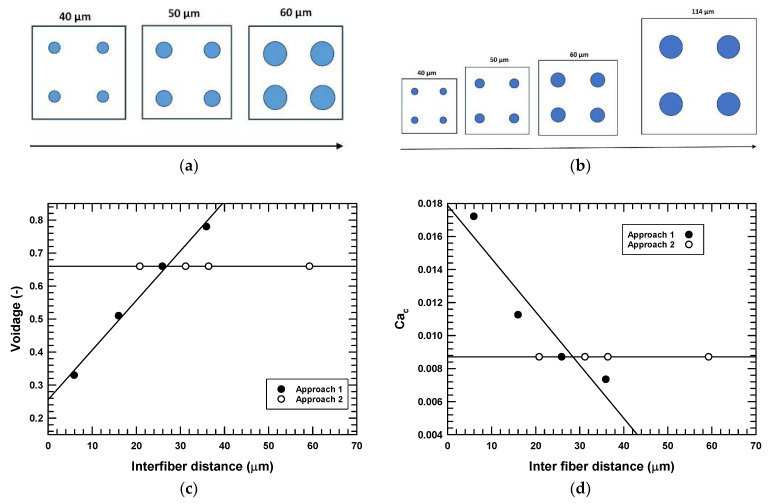
Comparison of approaches to packing the fibers into the fiber reactor and inter-fiber distances. (**a**) “CD” method, varying the fiber diameter but not the representative volume element dimensions, and (**b**) “CV” method varying the fiber diameter and the cell dimensions for constant voidage; (**c**) the voidage is plotted as a function of the inter-fiber distance; (**d**) effect of inter-fiber distance on capillary number.

The relationship between the voidage and the inter-fiber distance is presented graphically in Figure 2 for the two methods. As the voidage increased, so did the inter-fiber distance in method “CD”, but in method “CV”, voidage was constant. Figure 2 also shows the variation of the capillary number of the oil phase with inter-fiber distance. The capillary number changes in the “CD” method because it depends on the fluid velocity, which depends on the RVE area at a given constant flow rate. However, in the “CV” method, the flow area is always constant; hence, the capillary number remains unchanged. Note that since the fluid volume changes between some cases, we have reported all calculated interfacial surface areas as “specific surface area” by normalizing it via the fluid volume in the RVE. This also allowed comparing four-fiber model results to those previously reported for the single-fiber case. To study the effects of packing density on the phase structures and interfacial areas, models were run using the parameters provided in Table 1.

### 2.2. Domain and Boundary Conditions

**Figure 3:**
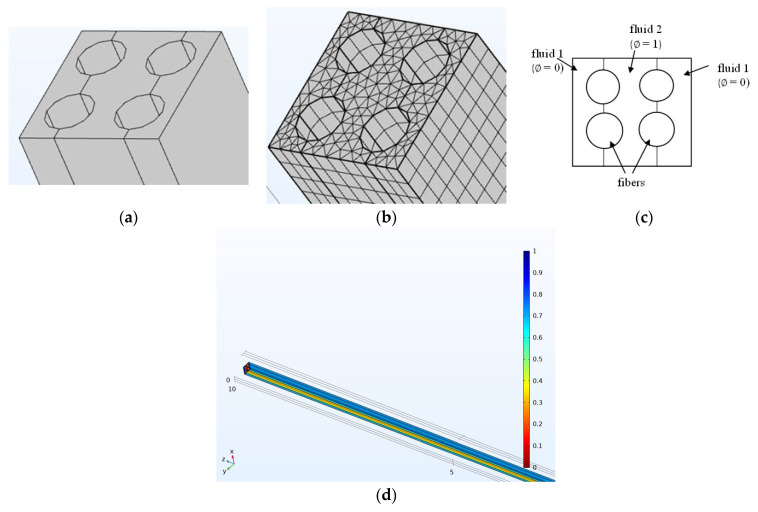
Schematic of (**a**) modeling domain at the inlet of the model showing the fluid volume and fibers, (**b**) meshed geometry, and (**c**) initial and inlet boundary conditions. Mid-planes drawn across the fibers in (**a**) and (**c**) are used to initialize the fluid phase boundaries (ϕ), (**d**): initialized phases of oil (red) and water (blue). The phases were initialized in a parallel manner adjacent to the fibers.

The inlet boundary and initial conditions are shown in Figure 3. The two phases were initialized by placing the fluid flows parallel to the fibers, with the oil phase in the inner and the water phase in the outer regions. The meshing sequence was created by meshing the inlet face with a triangular mesh and sweeping that inlet face down the entire domain.

A commercial fiber reactor for vegetable oil refining, using corn oil and water, was used as the target application. Water is represented by the subscript “w” and corn oil by the subscript “o”. Table 2 presents the physical properties of the corn oil and water phases. For all studies except those for which the contact angle was the independent variable, a wetted wall boundary condition with a contact angle of 10° was imposed on the fiber walls. This choice was made based on Santos et al. [26], where corn oil made a contact angle of 10° on stainless steel in supercritical CO_2_ applications. Symmetry boundary conditions were imposed on the outer boundaries of the RVE. The symmetry boundary condition was a semi-restricted flow condition discussed extensively in a previous study [21]. The velocities of the water and corn oil entering the RVE were selected to represent the experimental conditions for a tessellated 1-inch diameter fiber extractor vessel. Atmospheric pressure was specified as the outlet boundary condition at the outlet of the RVE.

### 2.3. Governing Equations and Solution Method

The Coupled Level Set (LS)–Volume of Fluid (VOF) method has been proven suitable for tracking the interface between the materials and their associated phases, with LS capturing the interface and VOF modeling different phases or materials. LS-VOF also has a track record of successfully modeling microscale flows within both laminar and turbulent regimes [27,28,29]. Thus, this study simulated oil and water interactions along microfibers using a coupled LS-VOF model provided via COMSOL Microphysics, a commercially available CFD tool (version 5.6 (build 280), 6.0 (build 405), and 6.1 (build 357)). The level set model solved the transport equation for the level set function  ϕ. The level set function (ϕ) was convected along with the flow using the same velocity field specified in the laminar flow interface. One set of the Navier–Stokes (N-S) equations was employed to solve the velocity, pressure, and viscous force fields for both phases. The fluid properties at the fluid–fluid interface were obtained using a weighted averaged function of the level set variable. The LS-VOF setup was identical to that used by the authors in their previous study of flow along a single microfiber [21], with the difference being that the present study explores water–oil interactions along four equidistant fibers, which would not affect the formulation of the governing equations and the numerical models applied to solve them. The specific equations are shown in detail in Supplemental Information.

### 2.4. Mesh Studies

The computational domain was discretized using a free tetrahedral mesh with a maximum element size of 10.9 μm, giving a total of 392,128 elements. Using the finite element method, the transient partial differential equations were converted to ordinary differential equations, and then from there, the temporal derivatives were discretized using the implicit Backward Differentiation Formula with a maximum order of 2 using a free time stepping scheme, resulting in Difference Algebraic Equations (DAEs). The average time step Δt used was about 100 μs. A linear iterative solver was employed to solve the DAEs iteratively until convergence was obtained. The convergence criterion was the relative tolerance and set at a value of 0.01. The model was run for 5 s with outputs printed every 0.1 s.

Meshing validation was performed to verify that the results were mesh-independent. Mesh convergence was performed for a study of the dilution of salt solution with pure water involving one fiber in an RVE. Table 3 and Figure S3 are summaries of the different meshing geometries modeled for mesh validation. The fine mesh and finer mesh were quite comparable. However, since the finer mesh took much more time to converge (up to one month), the fine mesh was employed.

## 3. Results and Discussion

In the following analysis, we first compare the one-fiber and four-fiber model results to understand their differences and whether a computationally simple one-fiber model is representative. We then present the inter-fiber distance, flow rate, and contact angle effects predicted when the neighboring fibers impact the various dependent variables such as phase structure, specific interfacial surface area, and pressure drop.

The channels formed by the fibers enclosed in a cm-scale pipe define a new type of pressure-driven microfluidic flow, here termed semi-restricted. Semi-restricted flow can be scaled to very high throughput. These boundary conditions, representing constraint only by the fiber(s) but without walls, contrast with a very new domain for microfluidics in the current literature termed “open channel microfluidics”. Open-channel flows are recently driven by electrophoresis, and the volumetric throughput may be enhanced by fabricating dozens of channels in parallel [30,31,32,33]. In contrast, the semi-restricted pressure-driven flows in FEs easily have tens of thousands of parallel channels. Thus, semi-restricted microfluidics has some similarities with open-channel microfluidics but is scalable to the separations and reaction needs of commodity volume production, including vegetable oil purification, petroleum desulfurization, vaccine production, nanoparticle production and separations for nuclear species [17,18,25,34,35,36]. The latter three fields are reported in the literature at the lab scale but are not yet commercial, to the author’s knowledge. Thus, the knowledge gap addressed here is using CFD to aid in the visualization of what types of phases develop in two-phase flows associated with microfluidic channels inside of FEs and how to control the surface area for extraction using inter-fiber distance, flow rate, and wetting or contact angle conditions. Note, however, that future work with fiber-packed modules can readily be modified to take advantage of the enhanced selectivity allowed by reactive fiber surfaces, such as is found in affinity chromatography (e.g., protein purification), or electrical fields, making the need for the base case of understanding pressure-driven flows even more compelling.

### 3.1. Single-Fiber Model and Four-Fiber Model Comparison

Including four fibers in the RVE instead of only one and then comparing results will illuminate how the nearest neighbor fibers might affect the flow and phase structures. A multi-fiber RVE also introduces the possibility of new independent variables: inter-fiber distance and fiber diameter. A base case was established, in common with the one-fiber model, using four 50 μm fibers with an inter-fiber distance of approximately 26 μm. To restate, four of the one-fiber models were tessellated into a larger unit cell, which was then termed the “four fiber” base case model, but with no boundary conditions inside of the new larger unit cell. Note that there was no difference in the initial conditions between the single- and four-fiber models. The flow rate of corn oil (Q_o_) in the compared cases was equal to the flow rate of water (Q_w_), i.e., Q_o_/Q_w_ = 1. The contact angle was 10°. Interfacial tension was 0.0316 N/m, and the corn oil and water viscosities were 0.0368 Pa.s and 0.001 Pa.s, respectively.

**Figure 4:**
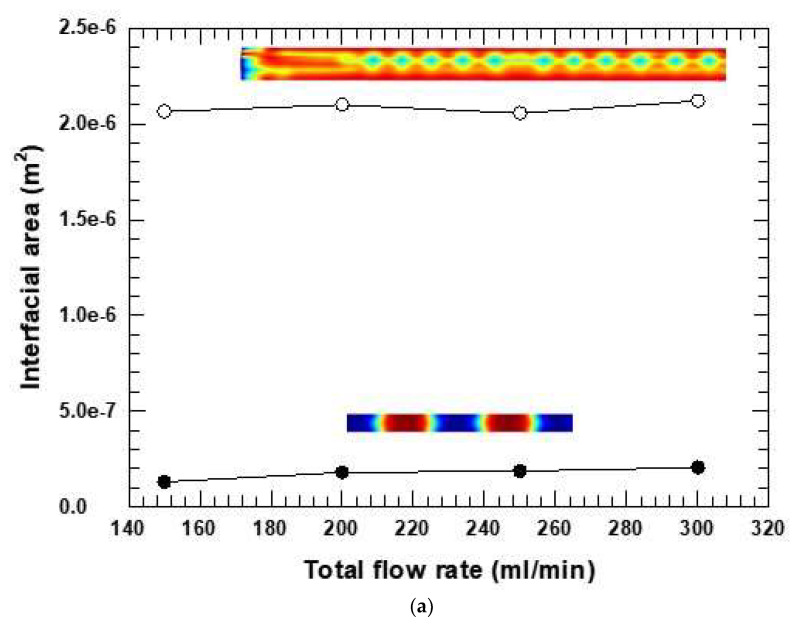

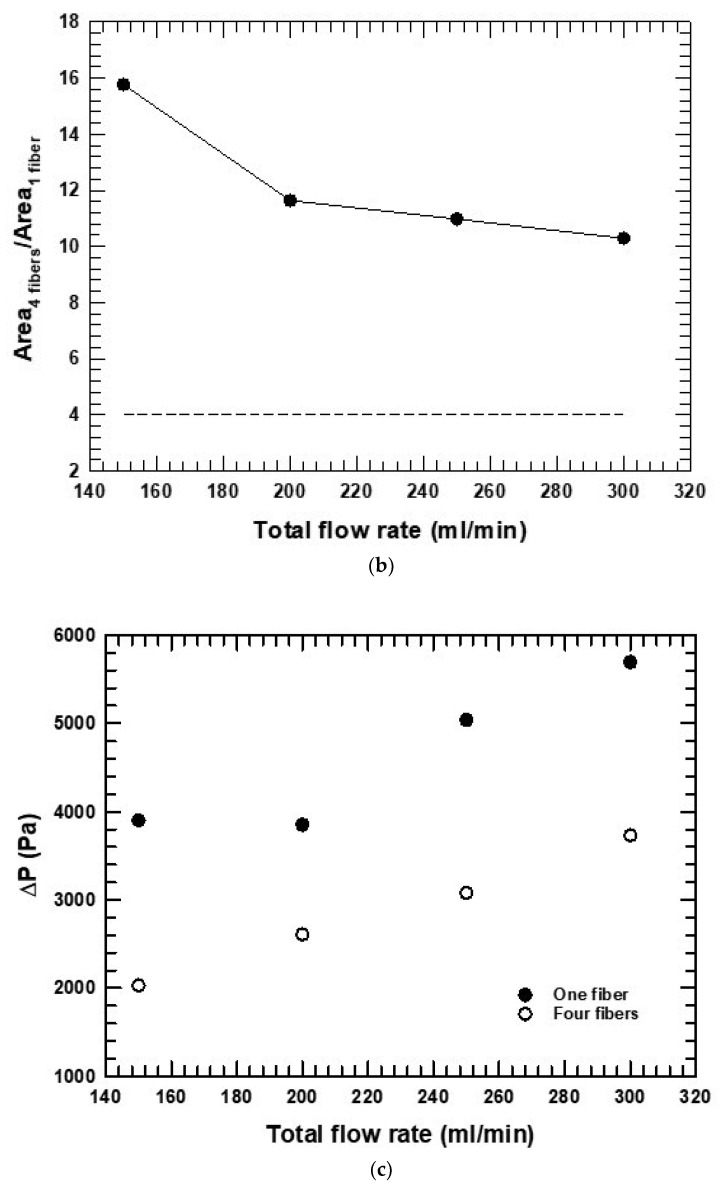
Comparison of single-fiber model and the four-fiber models using the total flow rate as a case study. (**a**) Interfacial areas vs. flow rate; (**b**) area scale factor from one fiber to four fibers; (**c**) pressure drop from one fiber to four fibers.

The graphs in Figure 4a show the results of the interfacial areas plotted against the total flow rate for the single- versus four-fiber cases. The insets show that the phase structures at a select flow rate differed, indicating that neighboring fibers did affect phase structure. Thus, radial flows must have been present in the four-fiber RVE and flows between channels interacted and inter-mixed.

The four-fiber RVE showed a much more complex phase structure in all cases, which could not be readily described by slugs or slug lengths, the most typical phase structure found in the microfluidic literature. Also, the interfacial surface area increased in the presence of the near-neighbor fiber effects. The predicted increase was not in the same proportion as the increased fluid volume (which is theoretically four times the increase in volume from the one-fiber RVE and shown by the dashed line in Figure 4b). In fact, the nonlinear increase in normalized interfacial surface areas was at least 2.5 times more than theoretical. The greater interfacial area, even normalized, was attributed to the neighboring effect of the fibers in the four-fiber model. Thus, the channels numbering into hundreds or thousands in an actual fiber reactor would be expected to create more complex flows and greater specific interfacial areas than the idealized one-fiber or even four-fiber RVE. Massingill et al. [18] and Kim et al. [19] confirm these predictions. The complexity of attaining static equilibrium by spontaneous imbibition on a set of parallel fibers in two-phase flows has been previously noted [28]. Related studies by Duprat et al. [37] and Prortiere et al. reported that the spreading of a static droplet of fluid on two fibers depends on the inter-fiber distance. Duprat et al. reported that when a drop of liquid was deposited between two parallel fibers at a fixed distance apart, the evolution of the liquid interface between the fibers depended on the ratio of the distance between the fibers (d) and radii of the fibers (r), i.e., d/r. At least three regimes of phase or droplet evolution were observed. The existence of three regimes of phase development dependent on inter-fiber distance indicates, importantly, that there could be one more flow regime beyond microfluidic versus pipe flow.

The Reynolds number (Re) in the present study was significantly small (Re << 1) and would not account for the phase complexity and mixing observed. Clearly, multidimensional flow paths were present in the fiber extractor. Hence, the fiber extractor might provide excellent guidance to one possible solution to the issues associated with microfluidic mixing [12]. Allowing creeping flows to interact may lead to localized mixing.

Figure 4c compares the pressure drops for single-fiber and four-fiber models as a function of the total flow rate. In both models, the pressure drop increased with the flow rate. However, at a given constant total flow rate, the pressure drop in the four-fiber model was at least 70% lower than in the single-fiber model, consistent with extra degrees of freedom for flow in the four-fiber case. Note that phase structures, previously reported as “slugs”, and which changed with the total flow rate, were quite different between the single-fiber and four-fiber cases. These different phase structures, however, did not affect the predicted pressure drop as significantly as did the total flow rate.

### 3.2. Effect of Inter-Fiber Distance on Phase Structures

The consideration of multiple fibers in the model introduces new possibilities for independent variables. As Duprat et al. [37] and Protiere et al. [38] mentioned, fiber diameter and inter-fiber distance might affect droplet structure, consistent with changes in interfacial surface area and pressure drop. These authors provided three different “dispersed” phase structures as inter-fiber distance increased in their studies, which they termed bridge, barrel, and column, if one considers the “matrix” phase to be air and the “dispersed” phase to be water. Different inter-fiber distances or packing geometries were therefore compared in the present study. For all configurations of fiber diameter and channel space considered here, the flow conditions were as follows: Q_w_ = 75 mL/min, Q_o_ = 150 mL/min (relative flow rate ratio, Q_o_/Q_w_ = 2), the interfacial tension, σ = 0.0316 N/m, the viscosities of the two phases, μ_o_ = 0.0368 Pa.s, μ_w_ = 0.001 Pa.s, respectively, and the contact angle, θ = 10°. One complication of varying packing density was that the fluid volumes were different across the cumulative data set; therefore, to compare the models, interfacial surface areas were divided by the fluid volumes to provide a specific interfacial area (m^2^/m^3^).

**Figure 5:**
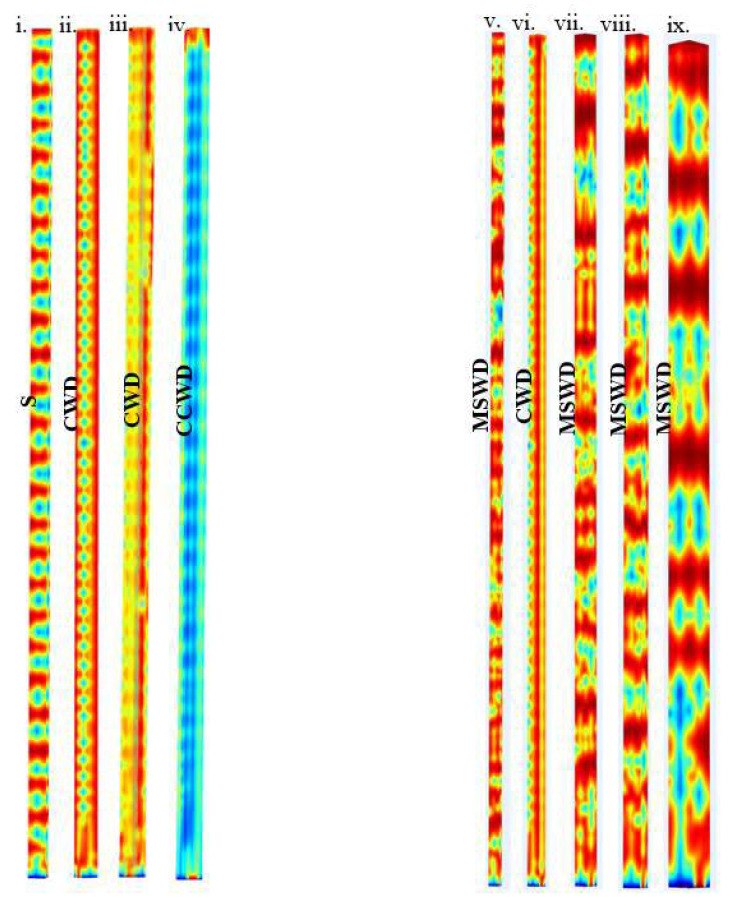
Scale is given by the diameter of the channel in microns, termed “W” below. Instantaneous phase structures generated from a four-fiber model using 40–114 μm diameter fibers as follows: (**i**) d_fib_ = 40 μm, W = 35.9 μm; (**ii**) d_fib_ = 50 μm, W= 25.9 μm; (**iii**) d_fib_ = 60 μm, W = 15.9 μm; (**iv**) d_fib_ = 70 μm, W = 5.9 μm; (**v**) d_fib_ = 40 μm, W = 20.8 μm; (**vi**) d_fib_ = 50 μm, W = 25.9 μm; (**vii**) d_fib_ = 60 μm, W = 31.2 μm; (**viii**) d_fib_ = 70 μm; W = 36.4 μm; (**ix**) d_fib_ = 114 μm; W= 59.6 μm. Figures ii and vi are the same, the base case. (**Left**) Models where RVE dimensions were constant, CD. (**Right**) Models where RVE void space was held constant, CV. Acronyms for phase structure: columnar with droplets (CWD), mixed slugs with droplets (MSWD), slugs (S), and core continuous with drops (CCWD).

The steady-state phase structures predicted by the models are shown in Figure 5i–ix. The predictions correspond to t = 5 s to cover at least one full residence time. Note that Figure 5i,iii,iv belong to one approach to varying packing density, the CD method (constant dimensions). The others are for an alternative approach, the CV method (constant voidage). When the RVE dimensions are constant, the inter-fiber distance changes with fiber diameter, as does voidage (CD). When the voidage is constant, the inter-fiber distance changes with fiber diameter, simultaneously changing RVE dimensions (CV). The phase structures noted were quite complex, not always slugs, with marked qualitative differences. To enable discussion, we characterized the various phases as columnar with droplets (CWD), mixed slugs with droplets (MSWD), slugs (S), and core continuous with drops (CCWD). Note that Figure 5ii,vi depict predictions using the same base case study, i.e., the same RVE. Generally, periodic wave-like phase structures were more evident for the smaller RVEs (on the left), whereas more complex ragged-edged slug-like phase structures were seen as the void space increased (on the right). Since the microfluidic literature generally describes slugs, drops, or films, these two different phase structures shown here support the hypothesis that at least three different flow regimes can exist within creeping flows in microfluidics.

**Figure 6:**
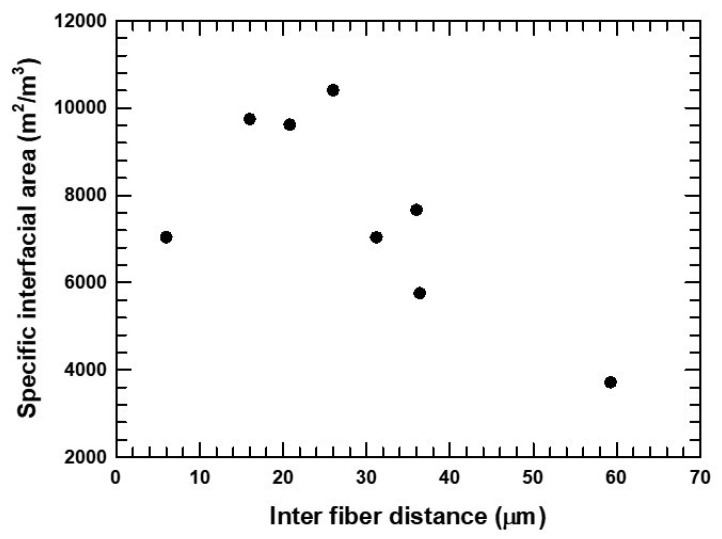
Effect of inter-fiber distance on the specific interfacial areas. Flow rate of corn oil Q_o_ = 150 mL/min, flow rate of water Q_w_ = 75 mL/min, relative flow rate Q_o_/Q_w_ = 2, contact angle = 10°, corn oil viscosity = 0.0368 Pa.s, interfacial tension = 0.0316 N/m.

**Figure 7:**
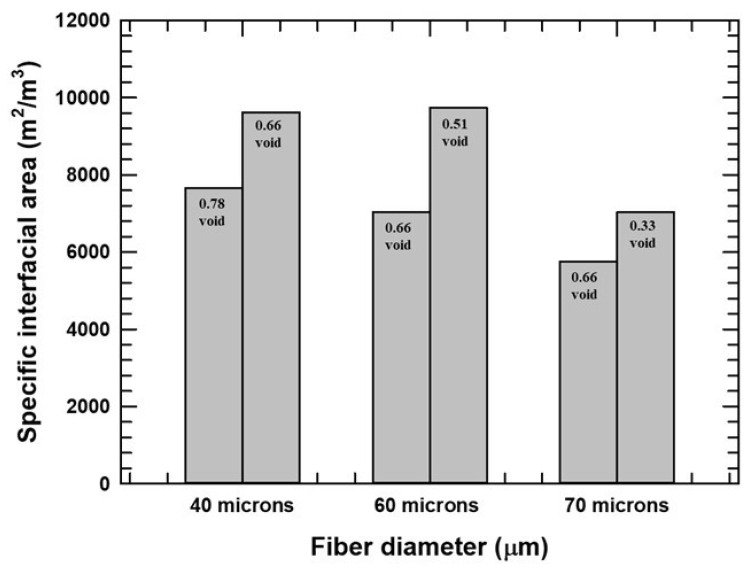
Specific area generated at constant fiber diameter and different voids. Voids left to right were 0.78, 0.66, 0.66, 0.51, 0.66, and 0.33.

Figure 6 shows the specific interfacial surface area against the inter-fiber distance for all the CD and CV models. The studies predicted an optimum inter-fiber distance for the generation of surface area, ranging between 10 and 50 μm. Since the surface area is essential for the overall mass transfer coefficients and the extraction efficiency of the vessel, this observation of an optimum is another key outcome of this CFD study. Figure 7 compares the effects of voidage or inter-fiber distance at a constant fiber diameter for three different fiber diameters. At constant fiber diameter, the more tightly packed the fibers, i.e., smaller void space, the greater the specific interfacial surface areas generated. An independent effect of fiber diameter may exist based on the trend for the 0.66 void space data as well.

Overall, the results show that the biggest predictor of interfacial surface area was the inter-fiber distance and that an optimal inter-fiber distance would exist (in terms of developing surface area) for a given set of feedstock, fluid properties, and process conditions.

**Figure 8:**
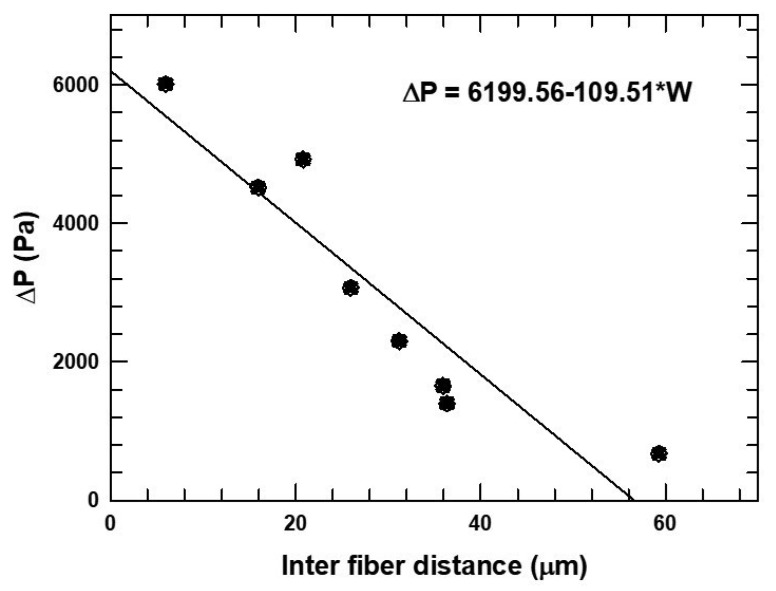
Effect of varying inter-fiber distance on the pressure drop. Inset is the effect of varying the hydraulic diameter on the pressure drop.

Figure 8 shows the pressure drop versus inter-fiber distance. Regardless of the type of phase structure (which was quite different for all of these cases), pressure drop generally decreased with wider channels, thereby lowering energy (pumping) requirements. Again, the contrasting phase structures observed and interfacial surface areas formed in each of the cases did not appear to have a large effect on pressure drop, which appeared to be governed mostly by the area available for flow or the wetted surface resisting flow.

### 3.3. Flow Rate Effect

Overall flow rate affects the phase structure in these two-phase fluids, and phase structure in turn affects the interfacial surface area available for mass transfer/extraction efficiency. Thus, having explored process conditions that affect phase structure, the focus now is the “bottom line”, which is how to connect to process efficiency. For a given overall volumetric flow rate, the relative flow rates of the two phases can also affect phase structure, again in turn yielding more or less surface area for extraction between phases. Thus, both overall and relative flow rates and their impact on specific interfacial areas were explored. Additionally, the overall flow rate was expected, of course, to affect pressure drop, one determinant of the energy efficiency of the process. CFD was used to predict pressure drop, but it can also uniquely predict whether, for a given overall flow rate, the phase structure alone affected the pressure drop. For example, in an extreme case, could formation of an emulsion with tiny droplets could increase the pressure drop.

The flow rate experiments were analyzed for 50 μm and 114 μm fiber diameters, the two extreme cases using the CV method. These two setups had a constant voidage of 0.66 but inter-fiber distances (W) of 26 μm and 59 μm, respectively. The number of fibers packed into a 1-inch reactor for the 50 μm diameter fibers would be approximately 87,000, and the number of fibers for a 114 μm diameter fiber would be approximately 17,000. Hence, there was a need to normalize the interfacial areas by fluid volume for comparison of output variables and ease of scale-up predictability. Total flow rates were varied from 100 mL/min to 300 mL/min. Relative flow rates were varied by keeping the flow rate of water constant at 75 mL/min and changing the flow rate of the corn oil from 45 mL/min to 125 mL/min. The contact angle was held at 10°, the interfacial tension, σ = 0.0316 N/m, and viscosity of corn oil and water, μ_o_ and μ_w_, were equal to 0.0368 Pa.s and 0.001 Pa.s, respectively. Intuitively, one would hypothesize that the pressure drop would be lower given wider clearances, but predicting how flow rates impacted interfacial surface areas would be more complex.

**Figure 9:**
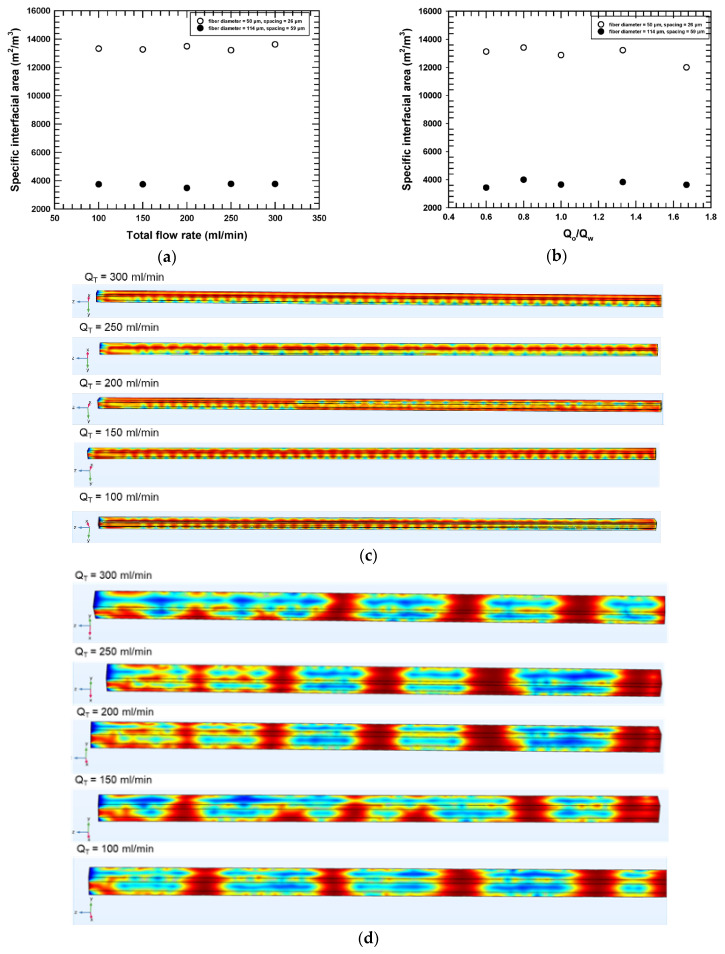
Effect of the flow rate on specific interfacial area in 50 μm fiber diameter with 26 μm inter-fiber distance and 114 μm fiber diameter with 59 μm inter-fiber distance models. (**a**) Total flow rate (relative phase ratio = 1). (**b**) Relative flow rate, 0.6 < Q_o_/Q_w_ < 1.8 where Q_w_ = 75 mL/min and contact angle = 10°. (**c**) Phase structures for distances of 26 µm and (**d**) phase structures for distances of 59 µm.

Figure 9a compares the plots of specific interfacial surface area as a function of the total flow rate at a relative flow rate of 1 for the two different geometries (26 µm and 59 µm inter-fiber distance). Figure 9b compares the plots of relative flow rate against the specific interfacial surface area for the two different geometries. Figure 9c,d show the phase structures for the total flow rates study when the distances were 26 μm (c) and 59 μm (d). The phase structure observed was unique to the RVE geometry regardless of the flow rates. In the 26 μm distance case, we observed periodic phase structures resembling the surface of a wave (CWD), whereas slugs with a unique ragged meniscus appeared (MSWD) for the 59 μm distance. Consequently, due to the uniqueness of phase structures developed for the two geometries, specific interfacial surface areas were almost constant for a given packing despite varying flow rates. However, the difference in the specific interfacial area was 2.5 times greater with the smaller (26 μm) clearance between fibers versus the larger clearance (59 µm) for a constant flow rate (Figure 9a,b). Note that the smaller clearance with the larger surface area falls within the predicted range of optimal inter-fiber distance described previously. To summarize, smaller clearances led to greater surface areas for extraction.

**Figure 10:**
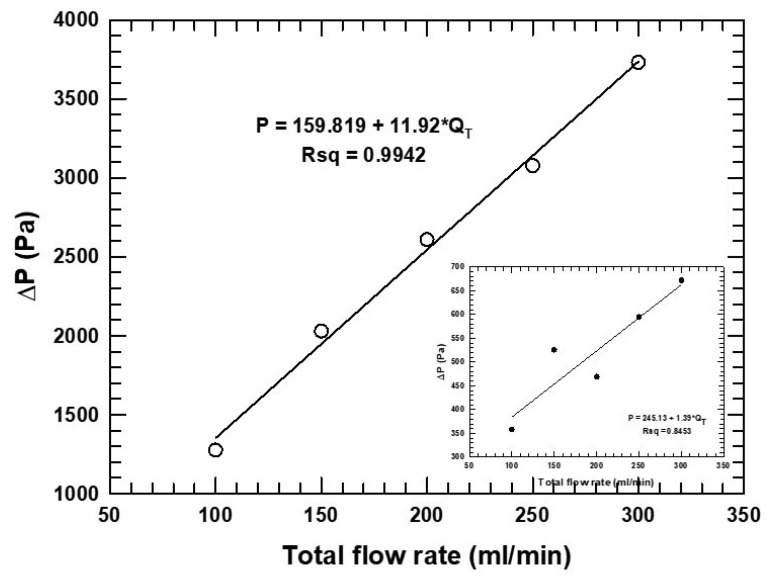
Effect of total flow rate on pressure drop for 50 μm with 26 μm. Inset shows 114 μm fiber diameter with 59 μm spacing.

**Figure 11:**
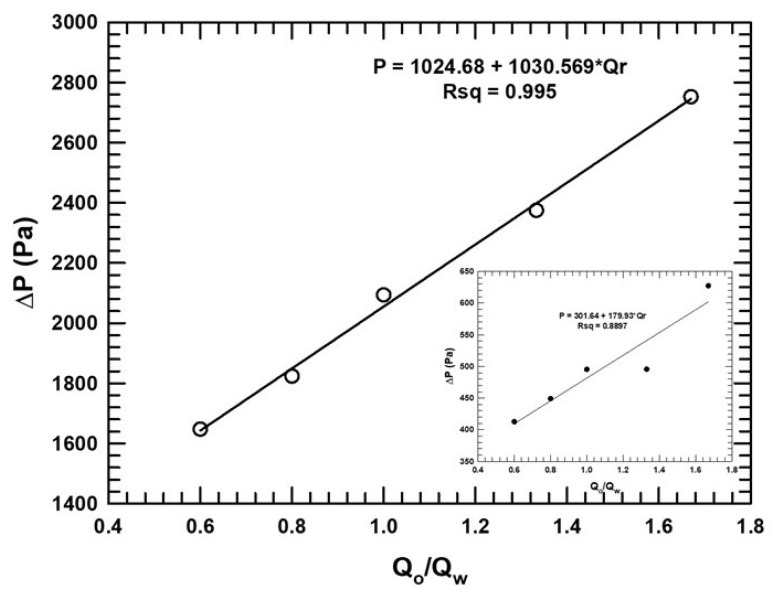
Effect of relative flow rate on pressure drop for 50 μm with 26 μm. Inset shows 114 μm fiber diameter with 59 μm spacing.

As the flow rate through constrained channels increases, it is well understood that pressure drop can be affected. Any increases in pressure drop can involve an energy cost when processing. In Figure 10 and Figure 11, the pressure drop is plotted against the flow rate for the two cases (the main graph represents the 26 μm distance, and the inset represents the 59 μm distance). Both graphs show an increase in pressure drop with flow rate; however, the slope is 10–20% less in the 59 μm case as the total flow rate of oil was increased. Thus, the sensitivity of the pressure drop to flow rate was greater for smaller clearances.

The overall flow rates also changed in the relative flow rate study. Hence, the effect of the overall flow rate might be a confounding variable. The sensitivity to the overall flow rate for the relative flow rate data set was plotted. Comparing two graphs, particularly their slopes, in Figure 10 to those in Figure S2 (see Supplementary Information) might tease out this potential artifact. The slopes were higher in both cases for the relative flow rate study (11.2 Pa.min/mL vs. 13.8 Pa.min/mL for the W = 26 µm case and 1.4 Pa.min/mL versus 2.4 Pa.min/mL for the W = 59 µm case). (Units of slope arise from calculating Δy/Δx in the graph.) Thus, the relative flow rate did affect pressure drop beyond overall flow rate changes in the study.

These model predictions were compared to the experimental work of Massingill et al. [18]. In their work, they utilized different fiber reactors with different packing geometries for the transesterification of triglyceride to biodiesel. Their results showed that a 100% conversion was achievable in a tube 30.5 cm in length and 10.9 mm in diameter packed with 570,000 fibers with only 18 mL of fluid volume at a total flow rate of 3.5 mL/min. In contrast, the conversion dropped to 98.8% when the same reactor was packed with fewer fibers (540,000 fibers) and operated at a higher total flow rate of 7 mL/min. The pressure in the first reactor was, however, higher as a result of the tighter clearances. The periodicity of the 26 μm inter-fiber distance phase structure in the present CFD results indicates behavior like a microfluidic channel even though it has four interacting (semi-restricted, semi-open) channels. The distinctive phase structures that were observed are similar to the three regimes observed as a consequence of static wetting between two parallel fibers separated by a given distance, as reported by Duprat et al. [37,38].

In conclusion, the flow rate effect on the specific interfacial area is not pronounced for a given packing geometry. However, the increase in specific interfacial surface areas becomes significant when the clearances are tighter, with an optimal inter-fiber distance noted. The confinement arising from the small clearances, however, does come with an energy (pumping) expense.

### 3.4. Wettability (Contact Angle) Effect

**Figure 12:**
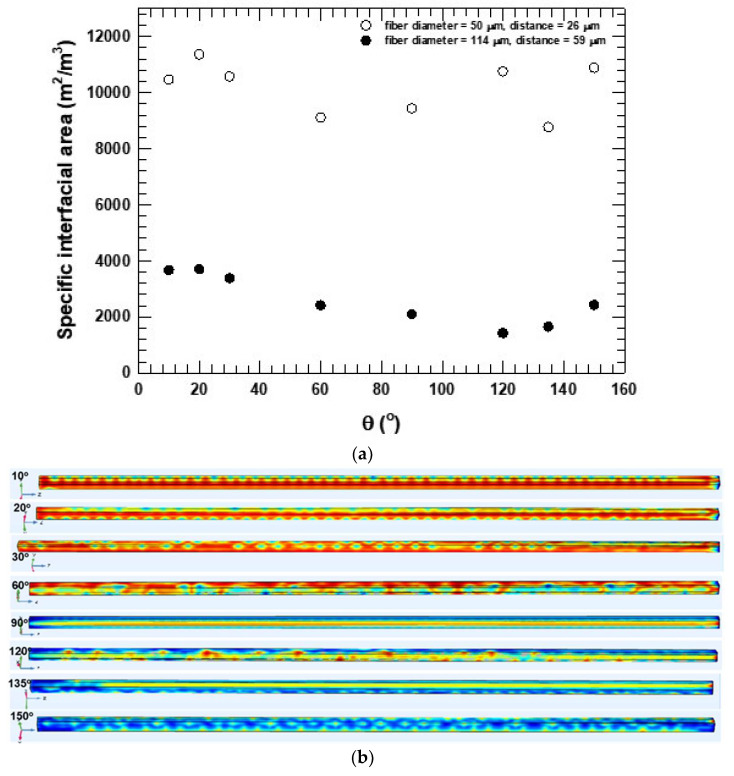

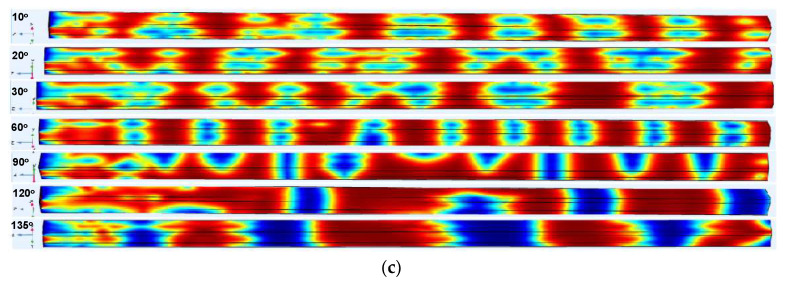
(**a**) Effect of contact angle on specific interfacial area for two cases: 50 μm diameter fiber with 26 μm distance between fibers (open circles) and 114 μm diameter fibers with 59 μm distance between fibers (filled circles). (**b**) Phase structures at different contact angles in the 50 μm diameter fiber geometry, W = 26 μm. (**c**) Phase structures at different contact angles in the 114 μm diameter fiber geometry, W = 59 μm. Note: both four-fiber RVEs have the same voidage.

**Figure 13:**
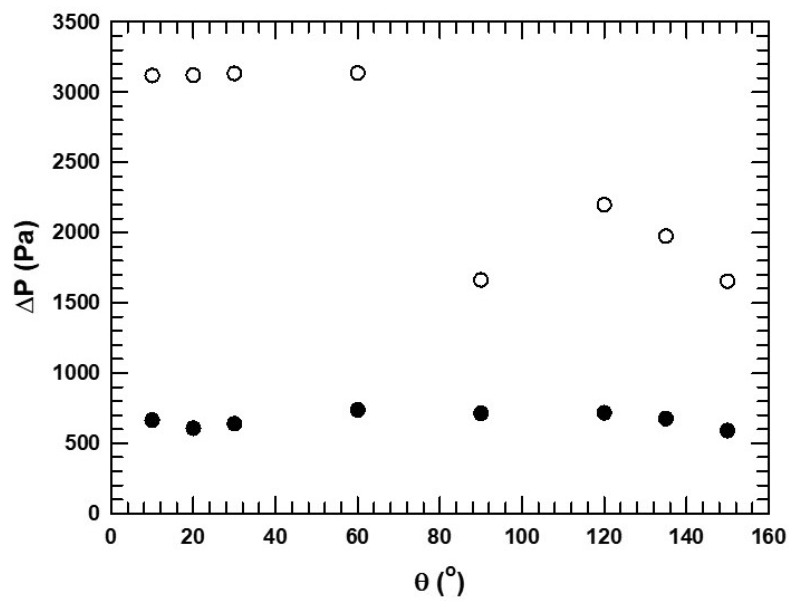
Effect of contact on pressure drop for two cases: 50 μm diameter fiber with 26 μm distance between fibers (open circles) and 114 μm diameter fibers with 59 μm distance between fibers (filled circles).

Estimating the surface tension force on the fiber walls involved specifying the two-phase contact angle on the fiber walls. The contact angle was defined as the angle between the fiber, the wetting, and the non-wetting phases. Two geometries were compared: a model with a fiber diameter d = 50 μm and inter-fiber distance W = 26 μm and a model with a fiber diameter d = 114 μm and inter-fiber distance W = 59 μm. The voidage in both models was 0.66. The contact angle varied from 10° to 150°. The fluid properties are those provided in Table 2. The effects of wettability are useful to explore the effects of the capillary action (capillary number, ${Ca}_{c}=v_{c}\mu_{c}/\sigma$) within each individual microchannel because the capillary and surface tension values are more significant than inertial and viscous forces. The capillary number for the contact angle study ranged from 7 × 10^−3^ to 1.7 × 10^−2^. As seen in Figure 2, the capillary number increased with decreasing inter-fiber distance. As the capillary number increased, the phase structures observed became more periodic and wave-like. Figure 12 compares the specific interfacial area for the two cases studied. Figure 12b,c indicate the representative phase structures observed. The phase structures changed significantly with the contact angle. For the W = 26 μm case (Figure 12b), the structures transitioned from regular periodic columnar with droplet structures at 10° and 20° to elongated double gyroid-like structures at 30° and to parallel type structures at 90°. The patterns transitioned further with increasing contact angle in reverse order from 120° all the way to 150°. We also note that the continuous phase underwent phase inversion when crossing the 90° angle. Thus, the oil phase acted as the wetting phase at lower angles, but after phase inversion, the water phase controlled spreading and was the wetting phase. For the W = 59 μm case (Figure 12c), the phase structures followed patterns with more irregular slug-like features with ragged edges. As the contact angle increased from 10°, the influence of fibers decreased. Hence, at angles greater than 90°, where the aqueous phase was the continuous phase, and the oil phase acted as the dispersed phase, the meniscus became rough-edged, and large slugs of both phases formed further downstream. Summarily, the contact angle effect revealed that more regular periodic phase structures were present in the case of the 26 μm distance between fibers, and more chaotic, random slug shapes were present at the larger distance, 59 μm. These different flow regimes were possibly a result of increasing clearance between fibers, resulting in smaller shear stresses and the added degree of freedom (DOF) for flows. The extra DOF suggests a third intermediate flow regime, resulting from conditions between microfluidic regimes and traditional pipe flow. Figure 12a, showing specific interfacial surface areas in the W = 26 μm case, confirms a transition relating to phase inversion. The system’s instability around phase inversion can be clearly seen in the plot (open circles in Figure 13). In the W = 59 μm case, the specific interfacial surface area curve follows an S-shape, again indicating the effects of phase inversion, but more stable. However, the magnitude of the specific surface areas produced was not strongly governed by the contact angle.

The effect of the contact angle on the pressure drop in both models and a graph of the pressure drop against the contact angle is shown in Figure 13. We observed two regimes for the 26 μm channels, shown by open circles. For the regime to the left of 90°, the corn oil phase was the wetting phase, and because corn oil is more viscous, the pressure drop was high. At angles beyond the inversion point, the pressure drop decreased because the low-viscosity aqueous phase was the wetting phase, and hence, there was less resistance against the flow. Again, the instability at phase inversion conditions can be noted. For the 59 μm distance case, the pressure drop was lower and not affected to any significant extent by phase inversion.

In summary, the oil phase was the wetting phase at smaller contact angles. As the contact angle increased, a phase inversion was observed. At a given contact angle, the specific interfacial surface area in the 26 μm clearances (d = 50 μm) was much larger than at the 59 μm clearances (d = 114 μm). Regardless of the wetting properties of the fiber, the most important factor governing the generation of interfacial surface area was the inter-fiber distance. Lower pressure drops were observed when water was in the wetting phase (at higher contact angles). Biofeedstock properties are, therefore, an important consideration for pressure drop and energy efficiency.

## 4. Conclusions

The fiber extractor (FE), a process vessel associated with process intensification for purification of biofeedstock, is a massively arrayed (i.e., numbered up) micro-structured extractor that provides several custom advantages of processing in a microfluidic environment. Such equipment offers a low footprint yet provides high throughput. The fluid flowed around the fibers, not through them. A CFD model of a four-fiber representative volume element was developed on a microfluidic scale. The effects of changing inter-fiber distance and process conditions on the phase structures, specific interfacial surface area, and pressure drop were presented. Predictions of the four-fiber CFD model include the following.

The presence of neighboring fibers clearly affected phase structure and indicated radial flows and mixing were present. Thus, the unique geometry of a fiber extractor has the potential for enhanced local mixing in microfluidic channels dependent on degrees of freedom for flow.

The interfacial surface area developed in the four-fiber case was more complex and proportionally much greater area than that of the idealized single-fiber annular flow, attributed to near-neighbor fiber effects.

The total flow rate, regardless of the type of phase structure, had the most significant effect on pressure drop.

The inter-fiber distance was the best predictor of the developed interfacial surface area. An optimum inter-fiber distance for generating surface area appeared to be between 10 and 50 μm.

The effect of contact angle (fiber wettability) on interfacial surface area and pressure drop was not pronounced. Contact angle contributed to the pressure drop by causing phase inversion. When the lower viscosity fluid (water) began to govern flow conditions, the pressure drop was also lower, particularly for the smaller clearances. However, at smaller inter-fiber distances, instabilities were noted during phase inversion, which could indicate processing instability.

Compared to the single-fiber case, the four-fiber model predicted new types of phase structures were noted such as standing waves and combinations of slugs and films.

At least three different flow regimes in creeping microfluidic flow were supported.

The phase structure in two-phase flows determines the interfacial surface area and, therefore, the potential for the extractor to enhance mass transfer and optimize extraction efficiency. These findings provide guidance for design and operation to optimize surface area and extraction efficiency by controlling the distances between fibers, the wetting or contact angle, and the flow rates. As the present work focused only on the unique fluidics possible in these microchannels, certainly CFD modeling of mass transfer, heat transfer, and interfacial reaction in two-phase flows with semi-restricted boundary conditions would extend the theoretical guidance available for these extractors. In addition, exciting work in open-channel microfluidics is recently reported with electrophoretic-driven flows, and extending fundamental modeling into other driving forces beyond pressure-driven flows could be relevant to some of the open-channel microfluidics work currently being reported.

## Figures and Tables

**Table 1:** Fiber packing models.

Method CD: Varied Inter-Fiber Distance by Changing Voidage and Keeping the Number of Fibers Constant (Voidage Is the Space Between Fibers Where Fluid Flows)			
Fiber Diameter (μm)	Voidage	Inter-Fiber Distance (μm)	Number of Fibers
40	0.78	36	87,700
50	0.66	26	87,700
60	0.51	15	87,700
70	0.33	5	87,700
**Method CV: Varied Inter-Fiber Distance by Keeping the Voidage Constant**			
**Fiber Diameter**	**Voidage**	**Inter-Fiber Distance (** **μ** **m)**	**Number of Fibers**
40	0.66	21	137,000
60	0.66	31	60,900
70	0.66	36	44,770
114	0.66	59	16,880

**Table 2:** Fluid properties and process conditions.

Density of water	1000 kg/m^3^
Density of corn oil	920 kg/m^3^
Surface tension @ temp = 35 °C	0.0316 N/m
Flow rate of water	45–150 mL/min
Flow rate of oil	75–150 mL/min
Viscosity of water	0.001 Pa.s
Viscosity of corn oil @ temp = 35 °C	0.0368 Pa.s

**Table 3:** Results of meshing validation studies.

	Normal	Fine	Finer
Number of elements	133,532	294,438	949,482
Minimum element size	3.9 × 10^−4^ m	1.96 × 10^−4^ m	7.83 × 10^−5^ m
Maximum element size	1.3 × 10^−3^ m	1.04 × 10^−3^ m	7.24 × 10^−4^ m
Average quality (skewness)	65.99	66.36	66.2

## Data Availability

The original contributions presented in this study are included in the article/Supplementary Materials. Further inquiries can be directed to the corresponding author.

## Supplementary Materials

The following supporting information can be downloaded at https://www.mdpi.com/article/10.3390/mi16040425/s1. Table S1: Calculation of area for flows; Figure S1: Comparing the influence of the external wall on fluid flow around the fiber and the influence of fibers on the fluid flow; Figure S2: Pressure drop versus total flow rate (where Q_w_ = 75 mL/min; Q_o_ = 45–125 mL/min); Figure S3: Comparison of changes in the concentration profile for different mesh sizes.
