# Supplementary material for: Effects of Fiber Arrangement on Flow Characteristics Along a Four-Fiber Element of Fiber Extractors
Source: Micromachines (Basel). 2025 Apr 1;16(4):425. doi: 10.3390/mi16040425 (PMC12029199; doi:10.3390/mi16040425)
Supplement: Supplementary file 1 [file micromachines-16-00425-s001.zip › micromachines-3477325-supplementary.pdf]

Supplementary Information

Effects of Fiber Arrangement on Flow Characteristics Along a Four-Fiber Element of Fiber Extractors

Oluwaseyi O. Ayeni [1], Holly A. Stretz [1]* and Ahmad Vasel-Be-Hagh [2]

[1] Department of Chemical Engineering, Tennessee Technological University, Cookeville, TN 38501, USA

[2] Department of Mechanical Engineering, University of South Florida, Tampa, FL 33620, USA

* Correspondence: hstretz@tntech.edu

This document contains: a table of variable definitions used in equations, calculations which led to the void area determination and number of fibers in a 1 inch pipe, the determination that the wall in a 1 inch packed pipe affected only 1% of the idealized flow in such a vessel, the ΔP plots along the Z axis length of the representative volume element (RVE) as contact angle was varied showing how the pressure varied at the interfaces of individual slugs, all of the data ΔP values plotted against slug count, and the plots comparing ΔP slopes as a function of total flow rate for the experiments where Qo/Qw were held constant and for those where only Q_w was held constant but relative flow rate was varied.

Fiber Extractor (FE)– Construction and working principle

We assumed that the fibers are arranged in a Cartesian (square) grid. The void area is obtained by multiplying the cross sectional area of the pipe with the void percentage. The steps in obtaining the number of fibers are as follows:

**Table S1.** Calculation of area for flows	
diameter of vessel	1 inch
cross sectional area	$Area = \frac{\pi \times 1^2}{4} = 0.078\ in^2 = 5.064 \times 10^8 \mu m^2$
void area	voidage = 66 % $Void\ area = 0.66 \times 5.064\ \times 10^8 = 3.3426\ \times\ 10^8$
area of a fiber	fiber diameter = 50 µm, $Fiber\ area = \frac{\pi \times 50^2}{4} = 1.963 \times 10^3 \mu m^2$
area occupied by fibers	$Total\ area\ occupied\ by\ fibers = (5.064 - 3.3426) \times 10^8$ $= 1.724 \times 10^8 \mu m^2$
total number of fibers in the pipe	$Total\ fibers = \frac{1.724\ \times 10^8}{1.963 \times 10^3} = 87{,}820\ fibers$
area of unit cell*	$Area\ of\ unit\ cell = \frac{Area\ of\ pipe}{total\ fibers} = \frac{5.064 \times 10^8}{87{,}820} = 5.76 \times 10^3 \mu m^2$
length of unit cell**	$length\ of\ unit\ cell = \sqrt{5.76 \times 10^3} = 75.94\ \mu m$

*Assume that a unit cell constitutes one fiber and a void area equal to the fiber area (i.e. 87,820 fibers gives 87, 820 voids); ** Assume a square unit cell

To quantify how large the walls affected flow, a simple calculation made to estimate how much of the actual flow is influenced by the walls of the channel showed that approximately only 1% of the area of flow was only influenced by the walls of the pipe. In this calculation, a unit cell located just adjacent to the walls of the larger pipe is considered (see figure S2), and the area of the annulus was compared to the area of the external walls.

$$rea\ of\ influenced\ by\ fibers = \frac{\pi}{4} \times (1in.^2 - (1in. - 150\mu m)^2)$$

$$= \frac{\pi}{4} \times (0.0254 - (0.0254 - 0.00015)^2) = 5.967 \times 10^{-6}$$

$$Fraction\ of\ flow\ area\ affected\ by\ wall\ = \frac{area\ inflieced\ by\ fibers}{area\ influenced\ by\ wall} = \frac{5.967 \times 10^{-6}}{5.067 \times 10^{-4}}$$

$$= 0.012\ or\ 1.2\%$$

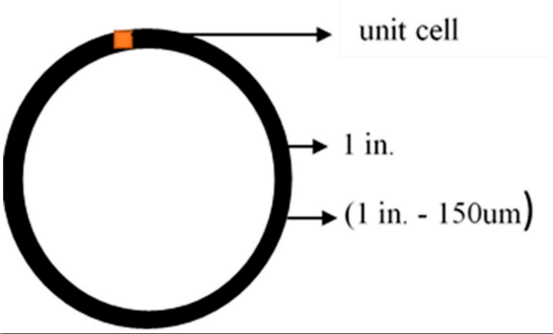

Figure S1. Comparing the influence of the external wall on fluid flow around the fiber and the influence of fibers on the fluid flow

Here the total area/volume of fluid affected by the pipe walls in a 1 inch packed vessel (bench scale) is estimated to be (for ideal flow) only 1% of the whole area/volume that the injected fluid experiences. Thus, the RVE of a single fiber with annular flow, semi-restricted boundary conditions, represents a significant amount of the environment fluid introduced into such a pipe would experience.

Governing Equations:

This section gives a full description of the equations (Equations 1 – 6) that were solved using the finite element method. The Multiphysics coupling interface called the Two-Phase Flow node coupled the Level Set and the Laminar flow interfaces. The level set equations solved the transport equation (Equation 2) for the level set function, $\emptyset$ where $\emptyset$ was advected along with the flow using the same velocity field specified in the Laminar flow interface in Equation 1. The parameter γ was a reinitialization parameter and ε_{ls} controlled the initial interface distance. To better describe $\emptyset$, this function was described for a given element (whether it was composed of, for example, oil, water or some mixture in between). In equation 2, the convective term is $\boldsymbol{u}.\nabla\phi$ and the diffusive term is $\nabla.(\epsilon_{ls}\nabla\phi)$.

The Navier-Stokes (N-S) equations, included under the Laminar flow interface in the program, solved a single equation for both phases (equation 1). The fluid properties (example viscosity and density) at the fluid-fluid interface were obtained using a weighted averaged function of $\emptyset$ (equations 4 and 5). In addition to the viscous forces, the momentum balance also solved for the interfacial tension force between the water and corn oil which is given in Equation 5 (where σ is the surface tension coefficient and κ is the average curvature) using the continuum surface force model described in Brackbill et al. (Brackbill, Kothe et al. 1992). Equations 6a and 6b are the physical parameters that model the convection of the fluid-fluid interface on the fiber walls. The contact angle θ_w is specified on the fiber wall, and the slip length, β is the distance from the wall where the velocity becomes zero (see Figure 3.4); the default value of mesh element size, 2.05 μm was used as the slip length in all cases. Equation 6c is added to the equation of the surface tension force, F_σ to account for the contact angle.

$$\nabla.\boldsymbol{u} = 0 \quad (1)$$

N-S equation

$$\left[\frac{\partial \boldsymbol{u}}{\partial t} + \boldsymbol{u}.\nabla \boldsymbol{u}\right] = -\nabla p + \mu(\nabla \boldsymbol{u} + (\nabla \boldsymbol{u})^T) + F_\sigma + \rho g$$

Level-set

$$\frac{\partial \phi}{\partial t} + \boldsymbol{u}.\nabla \phi = \gamma \nabla.\left(\epsilon_{ls}\nabla \phi - \phi(1-\phi)\frac{\nabla \emptyset}{|\nabla \emptyset|}\right) \quad (2)$$

Symmetry equations

$$\boldsymbol{u}.\boldsymbol{n} = 0 \quad (3)$$

$$K_n - (K_n.n)n = 0, K_n = Kn$$

Fluid properties

$$\rho = \rho_1 + (\rho_2 - \rho_1)\emptyset \quad (4a)$$

$$\mu = \mu_1 + (\mu_2 - \mu_1)\emptyset \quad (4b)$$

Surface tension

$$F_{st} = \sigma\delta\kappa n_{int} + \delta \nabla_s \sigma \quad (5)$$

$$\kappa = -\nabla.n_{int} = 1/R; \quad \nabla_s = (I - n_{int}n_{int}^T)\nabla$$

Wetting condition

$$u.n = 0 \quad (6a)$$

$$K_{nt} = \sigma\delta(n.n_{int} - \cos\theta_w)(n_{int}.t)t - \frac{\mu}{\beta}u_{slip} \quad (6b)$$

$$K_{nt} = K_n - (K_n.n)n, \ K_n = Kn, \quad \beta = f_h h_{min}$$

$$u_{slip} = u - (u.n)n \quad (6c)$$

$$F_\theta = \sigma\delta(n_{wall}.n - \cos\theta_w)n$$

All variables are defined below:

Definition of variables

**Nomenclature**	
a	half of inter-fiber distance, μm
W	inter fiber distance, μm
$\boldsymbol{u}$	velocity , ms^{-1}, (equation 1)
P	pressure, Pa, (equation 1)
$\mathbf{F_\sigma}$	surface tension force, Nm^{-3}, (equation 1)
R	Radius of curvature, m, (equation 5)
**g**	gravitational force, = 9.8 ms^{-2}, (equation 1)
n	interface normal, [-]
Kn	viscous stress tensor term, (equation 3 & 6b)
h_{min}	minimum mesh element length, m, (equation 6b)
f_h	factor of minimum element length, m
F_θ	wall force, Nm^{-2}
Knt	tangential viscous stress tensor, Nm^{-2}
**Greek Symbols**	
γ	re-initialization parameter, ms^{-1}, (equation 2)
ε_{ls}	parameter controlling initial interface distance, m, (equation 2)
φ	level set function, [-], (equation 2)
σ	interfacial tension coefficient, Nm^{-1}
κ	curvature, 1/m, (equation 5)
μ	fluid viscosity, $kgms^{-1}$
ρ	fluid density, kgm^{-3}
θ_w	contact angle at the wall, deg , (equation 6b)
β	slip length, m , (equation 6b)
**Subscripts**	
1, 2	fluids 1 and 2

Effect of relative flow rate on pressure drop: Qw = 75 ml/min, Qo = 45 – 125 ml/min. The graph is plotted as pressure drop versus total flow rate.

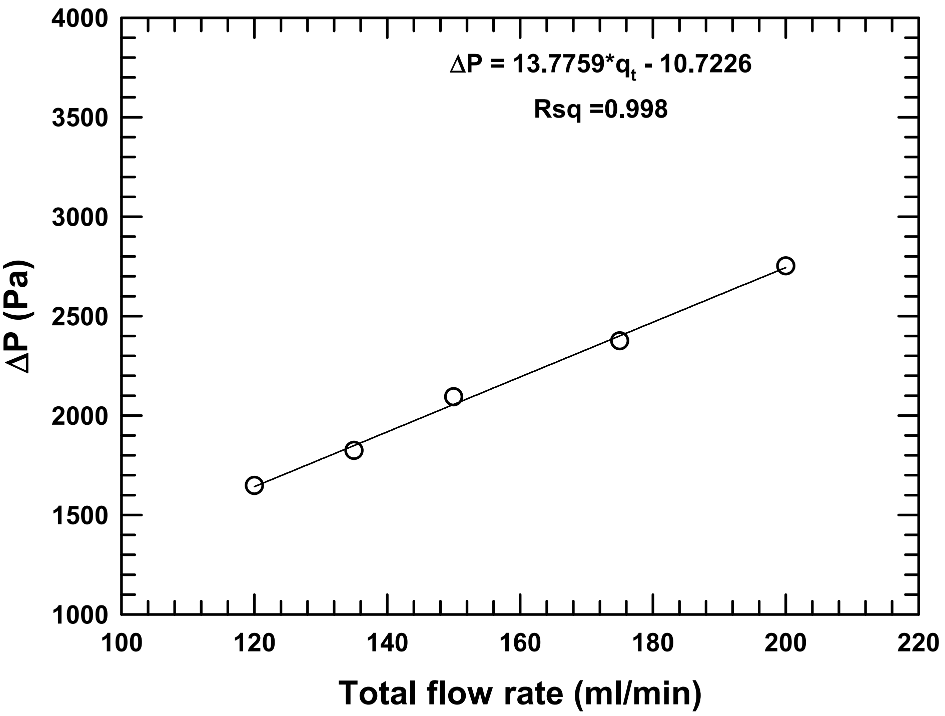
ΔP = 13.7759*q_t - 10.7226
Rsq =0.998
4000
3500
3000
2500
2000
1500
1000
100
120
140
160
180
200
220
ΔP (Pa)
Total flow rate (ml/min)

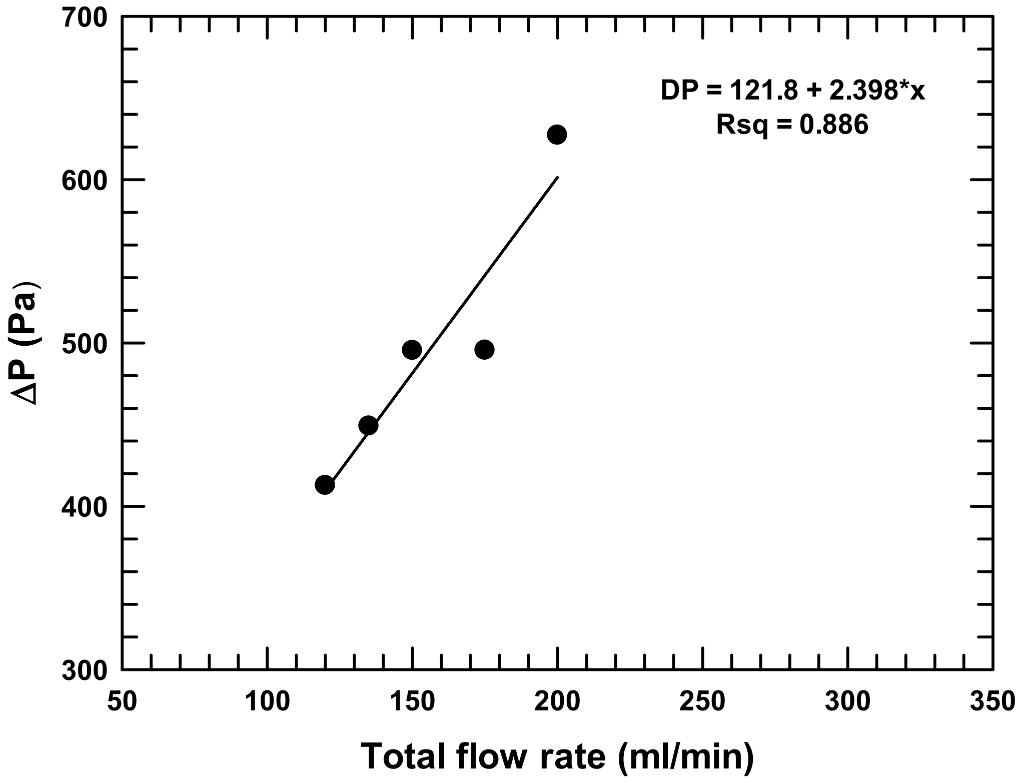

Figure S2. Pressure drop versus total flow rate (where Qw = 75 ml/min; Qo = 45 – 125 ml./min). (Top) When inter-fiber distance, W = 26 μm, fiber diameter = 50 μm. (Bottom) When inter-fiber distance, W = 59 μm, fiber diameter = 114 μm

More on Meshing Studies:

As discussed, the results of the mesh validation is presented in Figure **S3**. The fine mesh and finer mesh are quite comparable. However, since the finer mesh takes considerably more time to mesh, the fine mesh was employed RVE models.

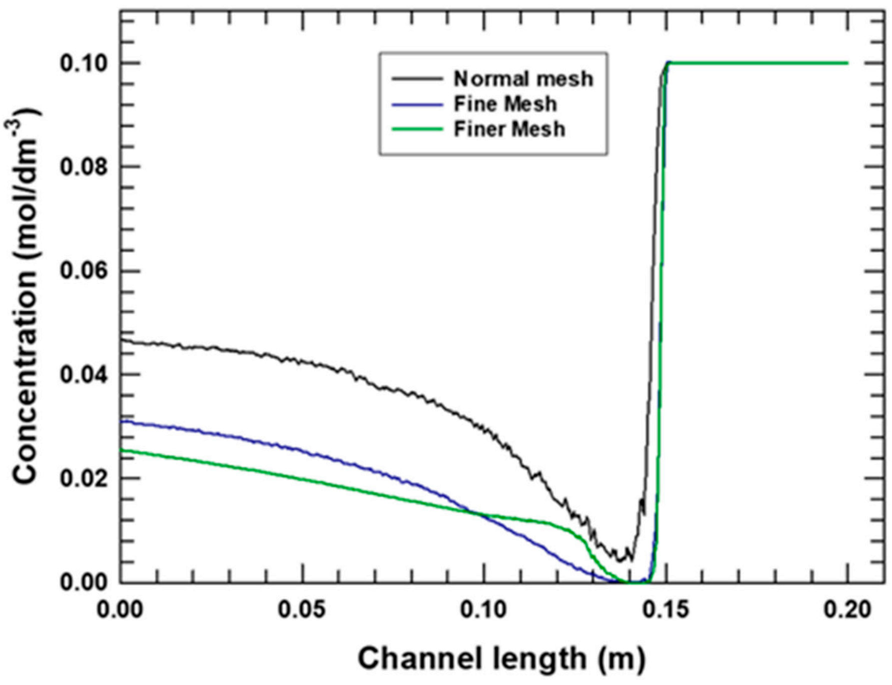

Figure S3. Comparison of changes in the concentration profile for different mesh sizes.
